# Subclinical thyroid diseases as a non-classical risk factor for cardiovascular diseases

**DOI:** 10.1590/1516-3180.2020.138230032020

**Published:** 2020-06-01

**Authors:** Isabela Martins Benseñor, Paulo Andrade Lotufo

**Affiliations:** I MD, PhD. Full Professor, Department of Internal Medicine, Faculdade de Medicina FMUSP, Universidade de Sao Paulo, São Paulo (SP), Brazil.; II MD, DrPH. Full Professor, Department of Internal Medicine, Faculdade de Medicina FMUSP, Universidade de Sao Paulo, São Paulo (SP), Brazil.

Cardiovascular diseases are the most common cause of death worldwide and in Brazil.^1^^,^^2^^,^^3^ Although mortality caused by these diseases has been decreasing over the last few decades, the pace of this decrease has differed according to socioeconomic status. The decrease has been greater among people of high socioeconomic status than among those of low socioeconomic status.^4^

The most common risk factors for cardiovascular diseases, i.e. hypertension, diabetes, dyslipidemia and smoking, do not explain all cases of cardiovascular diseases. As an example, in recent data from the Brazilian Longitudinal Study of Adult Health (ELSA-Brasil), these classical cardiovascular risk factors explain less than 40% of all of the burden of subclinical atherosclerosis, as measured from the common carotid artery intima-media thickness.^5^ However, assessments of these classical risk factors in other cohort studies have explained even less of the burden of atherosclerosis than in ELSA-Brasil. Rundek et al. reported that the classical risk factors explained around 11% of the variance of carotid intima-media thickness, and that addition of less traditional risk factors to the models only increased this proportion to 16%.^6^

Cardiovascular diseases are preventable, but control over the associated risk factors begins with identification of new risk factors beyond the classical ones. Several new risk factors have emerged over the last few years. Changes to thyroid function, such as those due to subclinical thyroid diseases or to alterations in thyrotropin (TSH) and free thyroxine (FT4) levels, are one of these. 

Thyroid diseases have worldwide prevalence, and Brazil is one of the countries with the highest prevalence of thyroid diseases.^7^ Although thyroid diseases are more common in women, and this is also true for Brazil, data from Brazilian studies have suggested that the female-to-male ratio for these diseases in Brazil is lower than in other countries.^8^^,^^9^

In addition to the burden of overt thyroid diseases, subclinical thyroid diseases characterized by low TSH levels with normal FT4 levels (as in the case of subclinical hyperthyroidism) or by high TSH levels with normal FT4 levels (as in the case of subclinical hypothyroidism) have been recognized over recent years as non-classical risk factors for cardiovascular diseases.^10^^,^^11^ Several studies have shown associations of subclinical hyperthyroidism and subclinical hypothyroidism with coronary heart disease,^12^^,^^13^ cardiovascular events^13^ and all-cause^12^ and cardiovascular mortality.^12^^,^^13^^,^^14^ A Brazilian study has also shown an association of subclinical thyroid diseases with all-cause and cardiovascular mortality, in a sample of Japanese-descendent Brazilians who were followed up for 7.5 years.^15^

Brazil has also contributed to studies on the association of subclinical thyroid diseases with subclinical atherosclerosis. Subclinical hypothyroidism was found to be associated with higher common carotid artery intimal-media thickness values, using euthyroid subjects as the reference (odds ratio, OR 1.30; 95% confidence interval, CI 1.06-1.59), after multivariate adjustment for sociodemographic and cardiovascular risk factors.^16^

In ELSA-Brasil, an association between low TSH levels (first quintile) and coronary artery calcium score was observed in the entire sample of men and women, even after multivariate adjustment for sociodemographic and cardiovascular risk factors (OR 1.57; 95% CI 1.05-2.35), using the third quintile as the reference. In an analysis restricted to men, TSH levels in the first quintile were associated with coronary artery calcium (CAC) scores > 100 Agatston units (OR 1.72; 95% CI 1.07-2.79). However, in women, there was a U-shaped curve such that TSH levels in both the first quintile (OR 3.31; 95% CI 1.31-8.37) and in the fifth quintile (OR 3.29; 95% CI 1.30-8.31) were associated with CAC > 100 Agatston units. Subjects with TSH levels within the range of subclinical hyperthyroidism and low-normal values (first quintile) had higher odds for CAC > 100, using the third quintile of TSH levels as the reference.^17^ Another study conducted in Brazil showed an association between subclinical hypothyroidism and CAC scores > 100 only among men older than 55 years, with a Framingham risk score > 10%.^18^

In this setting, it is essential to be able to diagnose occurrences of thyroid diseases in men. Previous Brazilian data showed that diagnoses of thyroid diseases among older men in low socioeconomic strata are made less often than would be expected.^8^ These individuals are also a high-risk group for cardiovascular diseases.^4^ Moreover, treatment for thyroid diseases is prescribed more commonly for women than for men, especially among people with low socioeconomic status.^8^^,^^9^ We can hypothesize that the high burden of thyroid diseases in men, which frequently remains undiagnosed and untreated, may form a non-classical risk factor for coronary heart disease and cardiovascular mortality in Brazil. On the other hand, we can hypothesize that some of the high burden of coronary heart disease in women may also be related to high frequency of thyroid diseases. Although thyroid diseases in women are more frequently treated than those in men, their occurrence is also influenced by low socioeconomic status. Data from ELSA-Brasil showed that women with high mean monthly income were more frequently treated than women with low income.^8^^,^^9^ Moreover, low socioeconomic status has also been shown to be a risk factor for lower rates of diagnosis and treatment of coronary heart disease, especially among women.^20^^,^^21^

This intricate relationship between thyroid and cardiovascular diseases, especially among people of low socioeconomic status in Brazil needs to be investigated more deeply to identify possible strategies for dealing with the problem. Brazil has particular characteristics that may suggest that considerable overlap exists between concomitant occurrences of these two diseases and presence of a high burden from their association in the general Brazilian population. Now is the right time to work on this!
